# Fungal Communities Associated with Peacock and Cercospora Leaf Spots in Olive

**DOI:** 10.3390/plants8060169

**Published:** 2019-06-12

**Authors:** Carla M.R. Varanda, Patrick Materatski, Miguel Landum, Maria Doroteia Campos, Maria do Rosário Félix

**Affiliations:** 1 ICAAM - Instituto de Ciências Agrárias e Ambientais Mediterrânicas, Instituto de Investigação e Formação Avançada, Universidade de Évora, Polo da Mitra, Ap. 94, 7006-554 Évora, Portugal; pmateratski@uevora.pt (P.M.); mdcc@uevora.pt (M.D.C.); 2 Instituto de Tecnologia Química e Biológica António Xavier, Universidade Nova de Lisboa, Av. da República, 2780-157 Oeiras, Portugal; miguel.landum@gmail.com; 3 Departamento de Fitotecnia, ICAAM - Instituto de Ciências Agrárias e Ambientais Mediterrânicas, Escola de Ciências e Tecnologia, Universidade de Évora, Polo da Mitra, Ap. 94, 7006-554 Évora, Portugal; mrff@uevora.pt

**Keywords:** biocontrol, fungal communities, ITS-PCR, *Pseudocercospora cladosporioides*, *Venturia oleaginea*

## Abstract

*Venturia oleaginea* and *Pseudocercospora cladosporioides* are two of the most important olive fungal pathogens causing leaf spots: peacock spot, and cercosporiosis, respectively. In the present study, fungal communities associated with the presence of these pathogens were investigated. Overall, 300 symptomatic and asymptomatic trees from different cultivars were sampled from Alentejo, Portugal. A total of 788 fungal isolates were obtained and classified into 21 OTUs; Ascomycota was clearly the predominant phylum (96.6%). Trees from cultivar ‘Galega vulgar’ showed a significant higher fungal richness when compared to ‘Cobrançosa’, which in turn showed significant higher values than ‘Picual’. Concerning plant health status, symptomatic plants showed significant higher fungal richness, mainly due to the high number of isolates of the pathogens *V. oleaginea* and *P. cladosporioides*. In terms of fungal diversity, there were two major groups: ca. 90% of the isolates found in symptomatic plants belonged to *V. oleaginea*, *P. cladosporioides*, *Chalara* sp., and *Foliophoma* sp. while ca. 90% of the isolates found in asymptomatic plants, belonged to *Alternaria* sp. and *Epicoccum* sp. This study highlights the existence of different fungal communities in olive trees, including potential antagonistic organisms that can have a significant impact on diseases and consequently on olive production.

## 1. Introduction

Olive (*Olea europaea* L.) is one of the most important fruit crops in countries characterized by Mediterranean climates and has been expanding in the last few years in producing countries and also to other countries such as Argentina, China, and India [1,2]. The growing awareness of the human health benefits associated with olive oil consumption is the main reason for this expansion. In Portugal, in addition to the high economic impact, olive has also significant environmental, social, and landscape impacts. The Alentejo region, benefiting from the biggest water reservoir in Western Europe built in the beginning of this century, the Alqueva dam, has rapidly seen the increase of super intensive olive orchards and is now responsible for the production of over 70% of the national olive oil. The Portuguese cultivars ‘Galega vulgar’ and ‘Cobrançosa’, and the Spanish cultivar ‘Picual’ are three of the most cultivated varieties, including in the Alentejo region, mainly due to the unique characteristics and properties of the olive oil they produce.

As in many other crops, the olive is susceptible to several pathogens that may affect the yield and quality of its products, with significant economic impacts [3,4]. Two of the most important and widespread fungal pathogens attacking the olive canopy are *Venturia oleaginea* and *Pseudocercospora cladosporioides*, responsible for peacock spot and cercosporiosis, respectively [5,6]. Both pathogens cause olive leaf spots, which are frequently difficult to distinguish [7]. Symptoms caused by *V. oleaginea* mainly start as sooty blotches on the upper surface of leaves that develop into dark green to black circular spots 0.2 to 1 cm in diameter, often surrounded by a yellowish halo around the spot [8,9,10]. Symptoms caused by *P. cladosporioides* in leaves are irregular chlorotic areas that turn necrotic in the upper surface and in the lower surface, grey areas corresponding to asexual fruiting structures [5,11,12]. In both cases, under favorable conditions, sunken brown lesions may appear on petioles and fruits. Trees affected by olive leaf spots caused by *V. oleaginea* and *P. cladosporioides* show defoliation of leaves and weakness or death of branches, resulting in reduced fruit set and a decrease in oil yield in the following years [13,14,15,16,17,18]. These fungi survive in leaf lesions and spread through conidia, by rain, wind, or insects, which is the main source of inoculum for primary infection in spring [19,20]. Both infections are associated with high humidity and low temperatures [7,19,21,22,23]. The establishment of several new super intensive olive orchards originates in a greater shading of the trees and favors the conditions for the development of these diseases. In highly susceptible cultivars, a complete loss of yield can occur under favorable climatic conditions. Currently, control measures are mostly based on chemical fungicides that are only partially effective if applied at the correct time and if disease risk is low; furthermore their frequent misapplication represent high costs and is harmful to human health and the environment [4,8,24]. According to the Directive 2009/128/EC, the European Model for Agriculture requires sustainable production systems and plant protection methods must obey the guidelines for integrated production of olives [25]. Studies concerning the evaluation of cultivars susceptibility to Cercosporiosis are scarce and many cultivars are considered as susceptible [26]. As for *V. oleaginea*, Roca and co-authors [27] determined the susceptibility of several cultivars under normal conditions, where ‘Galega vulgar’ was considered resistant, ‘Cobrançosa’ was moderately susceptible and ‘Picual’ was highly susceptible [5]. However, the recommendation of the use of resistant cultivars is difficult to follow by the producers, as they do not want to lose the specific quality and yield characteristics of a given cultivar. In addition, complete resistance is sometimes difficult to achieve. Thus, there is a need to develop alternative and effective control strategies that are safe for humans and the environment. In that sense, the study of microorganisms associated with specific pathogens will facilitate the understanding of their complex interactions, including the finding of potential antagonists that may be explored for designing new effective biological strategies for the control of the diseases, resulting in more sustainable systems. Although available data on fungal communities inhabiting the olive phyllosphere is still lacking, in the last few years there has been an increased interest on their study, due to the potential use of these fungi as biocontrol agents [28,29,30]; however little is known about the associations they establish with known pathogens. 

This work aimed to investigate the richness and diversity of fungal communities associated with symptomatic olive plants, with leaf spots caused by *V. oleaginea* and/or *P. cladosporioides*, and asymptomatic olive plants, without apparent symptoms. In addition, fungal communities associated with three olive cultivars with different susceptibilities to these diseases were evaluated, namely ‘Galega vulgar’, ‘Cobrançosa,’ and ‘Picual’. Consequently, it was hypothesized that the health status and type of cultivar could contribute to the differences in the fungal communities in terms of richness and diversity. The following research questions were addressed: Do the fungal richness and diversity vary (i) according to the different health status and (ii) among the different olive cultivars? This knowledge will contribute in determining the fungal strains that may naturally present an antagonistic effect against these pathogens and consequently will contribute to obtain more sustainable systems for plant production, such as the management of host-microbe interactions for improvement of plant protection and consequently crop yields.

## 2. Results

### 2.1. Fungal Identification—Structural Diversity

A total of 300 olive trees sampled (100 trees x 3 cultivars), half presenting leaf spots and half with apparently no symptoms, allowed the identification of 788 fungal isolates, either from fungal isolation in vitro or from direct leaf DNA. All fungi generated ITS amplification products with size ranging from 500 to 700 bp that were assigned to 21 OTUs: *Alternaria* sp., *Arthrinium* sp., *Aspergillus* sp., *Aureobasidium* sp., *Botrytis* sp., *Bullera* sp., *Chaetomium* sp., *Chalara* sp., *Cryptococcus* sp., *Diaporthe* sp., *Epicoccum* sp., *Erythrobasidium* sp., *Fusarium* sp., *Fusicladium* sp., *Neofusicoccum* sp., *Nigrospora* sp., *Foliophoma* sp., *P. cladosporioides*, *Saccharata* sp., *V. oleaginea* and *Sporobolomyces* sp. All isolates were identified to genus level with >97% identity. *V. oleaginea* and *P. cladosporioides* were identified to species level, with 100% identity to sequences in the GenBank (Accession number AF338403.1 for *V. oleaginea* and MH856296.1 and AY438248.1 for *P. cladosporioides*). The same OTU within a sample was considered as a single isolate. 

The amplification of total DNA from fungal cultures, allowed the identification of 11 OTUs and the amplification of total DNA from leaf tissues, allowed the identification of 19 OTUs. *Fusarium* sp. and *Nigrospora* sp. were identified only when fungal cultures were used. On the other hand, *Arthrinium* sp., *Bullera* sp., *Chaetomium* sp., *Chalara* sp., *Cryptococcus* sp., *Erythrobasidium* sp., *Neofusicoccum* sp., *Sporobolomyces* sp., *P. cladosporioides,* and *V. oleaginea* did not grow in vitro and were only identified when DNA from leaf tissues was used.

Overall, nearly all isolates obtained belong to Phylum Ascomycota (96.6%), represented by four classes, with the class Dothideomycetes the most representative (70.2%), followed by Leotiomycetes (14.5%), Sordariomycetes (10.0%), and Eurotiomycetes (1.9%). An amount of 3.4% of the isolates belongs to Phylum Basidiomycota, represented by three classes, class Tremellomycetes (2.3%), Microbotryomycetes (0.63%), and Cystobasidiomycetes (0.51%). Seven genera *Venturia* (16.0%), *Alternaria* (15.0%), *Epicoccum* (13.8%), *Pseudocercospora* (13.2%), *Chalara* (11.0%), *Foliophoma* (8.4%), and *Botrytis* (3.4%) together comprised nearly 80% of the fungal diversity found.

Univariate and multivariate analyses were performed to detect significant differences in total richness in the endophytic fungi in the two factors “Health status” and “Cultivar”. SIMPER analysis on asymptomatic data revealed that only three fungal OTUs *Alternaria* sp. (62.8%), *Epicoccum* sp. (29.1%), and *Arthrinium* sp. (2.1%) represent 94.0% of the similarities (Table 1).

SIMPER analysis on symptomatic data revealed that four fungal OTUs *V. oleaginea* (40.4%), *P. cladosporioides* (27.1%), *Chalara* sp. (17.0%) and *Foliophoma* sp. (9.5%) represent 94.0% of the similarities (Table 1). In addition, SIMPER analysis revealed that only three OTUs *Alternaria* sp., *Epicoccum* sp. and *Aureobasidium* sp. were present in both symptomatic and asymptomatic olive trees. Twelve OTUs *Botrytis* sp., *Bullera* sp., *Chalara* sp., *Cryptococcus* sp., *Erythrobasidium* sp., *Fusicladium* sp., *Neofusicoccum* sp., *Foliophoma* sp., *P. cladosporioides*, *Saccharata* sp., *V. oleaginea*, and *Sporobolomyces* sp. were exclusive of the symptomatic olive leaves and six OTUs *Arthrinium* sp., *Aspergillus* sp., *Chaetomium* sp., *Diaporthe* sp., *Fusarium* sp., and *Nigrospora* sp. were exclusive of the asymptomatic olive leaves. SIMPER analysis on ‘Galega vulgar’ revealed seven fungal OTUs: *Alternaria* sp. (26.94%), *Epicoccum* sp. (25.95%), *V. oleaginea* (12.46%), *Chalara* sp. (10.68%), *Foliophoma* sp. (9.34%), *P. cladosporioides* (7.75%), and *Botrytis* sp. (2.83%) that represent 95.9% of the similarities (Table 1). On ‘Cobrançosa’, data revealed the presence of five fungal species: *Alternaria* sp. (33.66%), *V. oleaginea* (20.06%), *Chalara* sp. (18.51%), *P. cladosporioides* (12.87%), and *Epicoccum* sp. (10.46%) that represent 95.5% of the similarities (Table 1). On ‘Picual’ five fungal OTUs *V. oleaginea* (26.17%), *Epicoccum* sp. (21.89%), *P. cladosporioides* (19.29%), *Alternaria* sp. (19.02%), and *Foliophoma* sp. (10.63%) represent 96.9% of the similarities (Table 1). At cultivar level, SIMPER analysis revealed that thirteen OTUs *Alternaria* sp., *Arthrinium* sp., *Aspergillus* sp., *Aureobasidium* sp., *Botrytis* sp., *Chaetomium* sp., *Chalara* sp., *Diaporthe* sp., *Epicoccum* sp., *Fusarium* sp., *Nigrospora* sp., *P. cladosporioides*, and *V. oleaginea* were present in ‘Galega vulgar’, ‘Cobrançosa’ and ‘Picual’. ‘Galega vulgar’ presented five OTUs exclusively; *Bullera* sp., *Cryptococcus* sp., *Fusicladium* sp., *Saccharata* sp., and *Sporobolomyces* sp. One OTU, *Neofusicoccum* sp. was exclusive in ‘Cobrançosa’ and one OTU *Erythrobasidium* sp. was exclusive in ‘Picual’.

### 2.2. Multivariate Data Analysis—Fungal Richness

**Figure 1:**
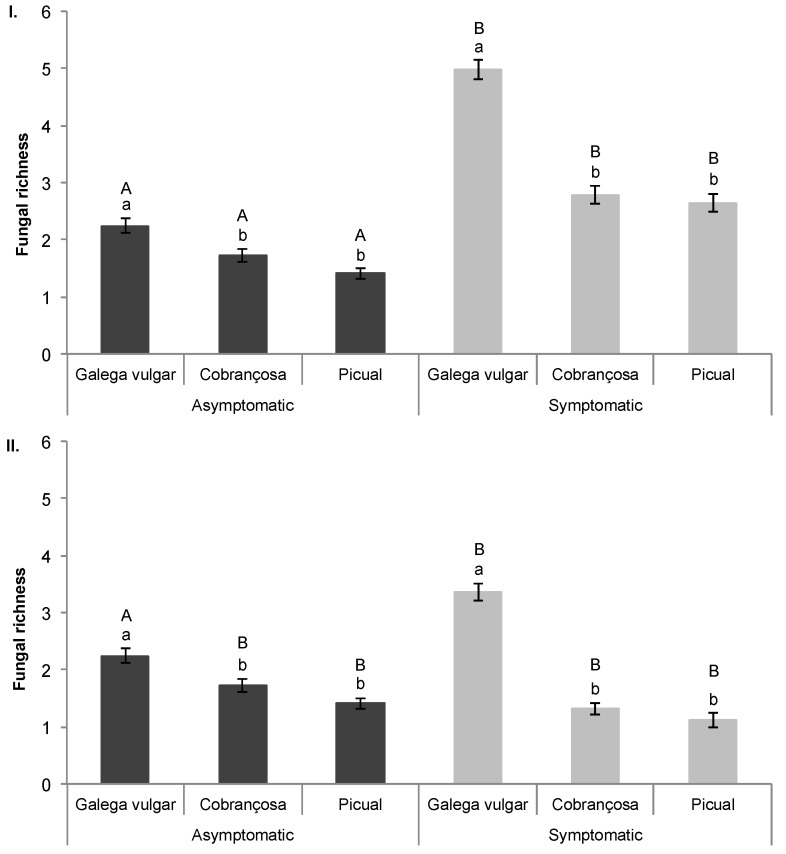
Mean fungal richness ± standard error (SE) at each: health status (asymptomatic and symptomatic) and cultivar (‘Galega vulgar’, ‘Cobrançosa’ and ‘Picual’). Different letters indicate significant differences (p < 0.05) and equal letters indicate no significant differences (p > 0.05). Lowercase letters indicate comparisons between cultivars (‘Galega vulgar’, ‘Cobrançosa’, and ‘Picual’) within each Health status (asymptomatic or symptomatic). Capital letters indicate comparisons between the same cultivar (‘Galega vulgar’, ‘Cobrançosa’, and ‘Picual’) in the different Health status (Asymptomatic and Symptomatic). (**I**) represents fungal richness dataset and (**II**) represents fungal richness dataset excluding both pathogenic fungi *V. oleaginea* and *P. cladosporioides*.

The mean fungal richness ± SE was 1.79 ± 0.07 in asymptomatic olive plants and 3.47 ± 0.13 in symptomatic (Figure 1-I). PERMANOVA analyses showed significantly higher fungal richness in symptomatic olive plants (Factor “health status”, p = 0.0166) (Table 2) when compared to asymptomatic plants.

**Figure 2:**
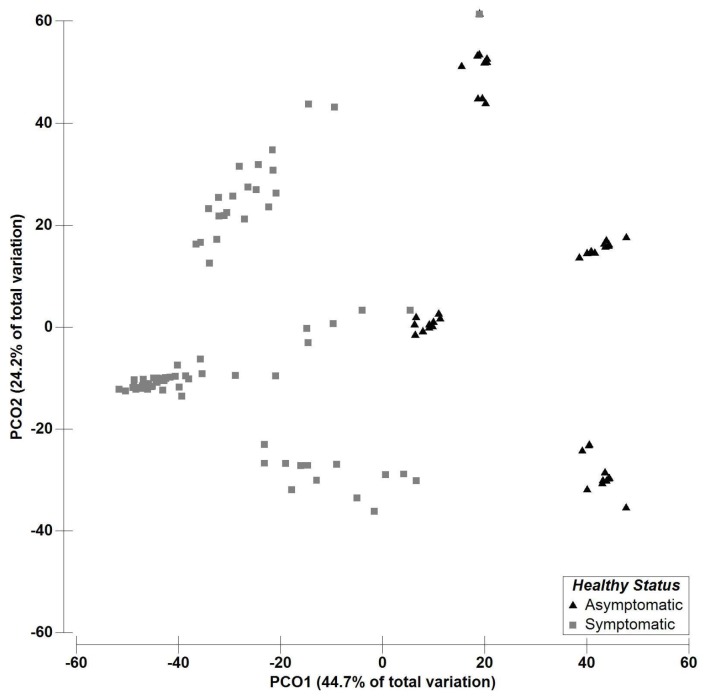
Principal coordinates analysis PCO based on the fungal richness dataset for the factor “Health status” Asymptomatic and Symptomatic (2 levels, fixed). PCO1 = 24.2% and PCO2 = 44.7%.

These results are supported by the PCO ordination plot and clearly reflect a distinct pattern for fungal richness between asymptomatic and asymptomatic olive plants. The PCO ordination of the endophytic richness showed that the first two components (PCO1, 44.72% and PCO2, 24.24%) accounted for 68.9% of the variability of the data (Figure 2).

The mean fungal richness ± SE excluding both pathogenic fungi *V. oleaginea* and *P. cladosporioides* was 1.79 ± 0.07 in asymptomatic olive plants and 1.93 ± 0.11 in symptomatic plants (Figure 1-II). In addition, PERMANOVA analyses were performed excluding both pathogenic fungi *V. oleaginea* and *P. cladosporioides*, showing no significant differences in fungal richness between symptomatic and asymptomatic (Factor “health status”, p = 0.5397).

**Figure 3:**
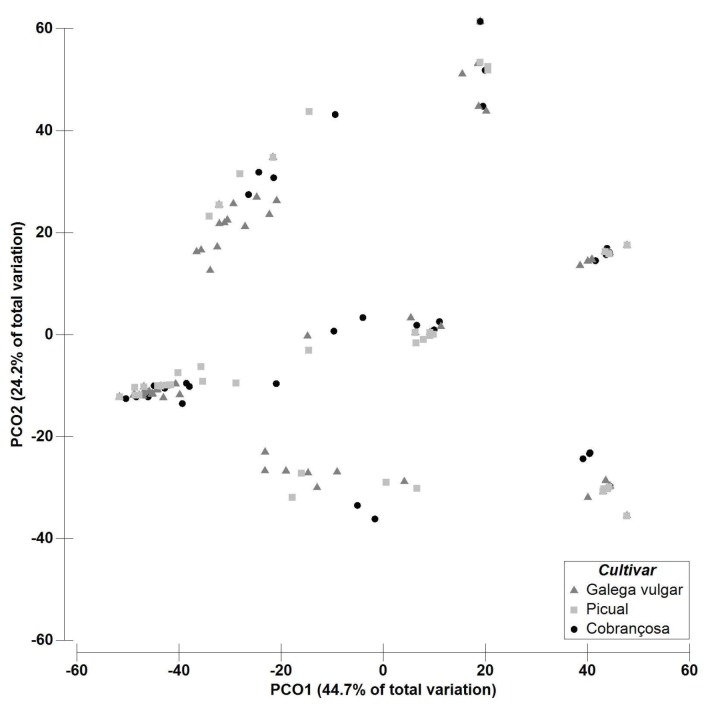
Principal coordinates analysis PCO based on the fungal richness dataset for the factor “Cultivar” ‘Galega vulgar’, ‘Cobrançosa’ and ‘Picual’ (3 levels, random). PCO1 = 24.2% and PCO2 = 44.7%.

At the cultivar level, the mean fungal richness ± SE was 3.61 ± 0.17 in ‘Galega vulgar’ followed by 2.25 ± 0.11 in ‘Cobrançosa’, and 2.02 ± 0.11 in ‘Picual’ (Figure 1-I). PERMANOVA analyses showed significant (p = 0.0001) higher fungal richness in ‘Galega vulgar’ when compared to ‘Cobrançosa’ and ‘Picual’ and no significant differences (p = 0.07) between these last two cultivars. These results are supported by the PCO ordination plot and clearly reflect a high variability for fungal richness between ‘Galega vulgar’ and the other cultivars. The PCO ordination of the endophytic richness showed that the first two components (PCO1, 44.72% and PCO2, 24.24%) accounted for 68.9% of the variability of the data (Figure 3).

At the cultivar level, the mean fungal richness ± SE excluding both pathogenic fungi *V. oleaginea* and *P. cladosporioides* was 2.80 ± 0.11 in ‘Galega vulgar’ followed by 1.52 ± 0.08 in ‘Cobrançosa’ and 1.26 ± 0.08 in ‘Picual’ (Figure 1-II). PERMANOVA analyses excluding both pathogenic fungi still showed significant (p = 0.0001) higher fungal richness in ‘Galega vulgar’ when compared to ‘Cobrançosa’ and ‘Picual’ and no significant differences (p = 0.3464) between these last two cultivars. Individual pairwise comparisons confirmed the high variability in terms of fungal richness, with consistent higher values in symptomatic plants compared to asymptomatic plants at each cultivar; ‘Cobrançosa’ (Pairwise Tests, p _symptomatic vs. asymptomatic_ = 0.0001), ‘Galega Vulgar’ (Pairwise Tests, p _symptomatic vs. asymptomatic_ = 0.0001) and ‘Picual’ (Pairwise Tests, _p symptomatic vs. asymptomatic_ = 0.0001). In symptomatic plants, individual pairwise comparisons between cultivars showed significant higher fungal richness in ‘Galega vulgar’ compared to ‘Cobrançosa’ (Pairwise Tests, p _‘Galega vulgar’ vs. ‘Cobrançosa’_ = 0.0001) and ‘Picual’ (Pairwise Tests, p _‘Galega vulgar’ vs. ‘Picual’_ = 0.0001), and no significant differences between ‘Cobrançosa’ and ‘Picual’ (Pairwise Tests, p _‘Cobrançosa’ vs. ‘Picual’_ = 0.5121). In asymptomatic plants, individual pairwise comparisons between cultivars also showed significant higher fungal richness in ‘Galega vulgar’ compared to ‘Cobrançosa’ (Pairwise Tests, p _‘Galega vulgar’ vs. ‘Cobrançosa’_ = 0.0021) and ‘Picual’ (Pairwise Tests, p _‘Galega vulgar’ vs. ‘Picual’_ = 0.0001), and no significant differences between ‘Cobrançosa’ and ‘Picual’ (Pairwise Tests, _p ‘Cobrançosa’ vs. ‘Picual’_ = 0.0583).

Individual pairwise comparisons, excluding both pathogenic fungi, showed that only ‘Galega Vulgar’ presented significant higher values of fungal richness in symptomatic plants when compared to asymptomatic plants (Pairwise Tests, p _symptomatic vs. asymptomatic_ = 0.0001), with ‘Cobrançosa’ and ‘Picual’ showing no significant differences (Pairwise Tests, p _symptomatic vs. asymptomatic_ = 0.2569 and Pairwise Tests, p _symptomatic vs. asymptomatic_ = 0.3437, respectively). In addition, when both pathogenic fungi were excluded, in symptomatic plants, individual pairwise comparisons between cultivars still showed significant higher fungal richness in ‘Galega vulgar’ compared to ‘Cobrançosa’ (Pairwise Tests, p _‘Galega vulgar’ vs. ‘Cobrançosa’_ = 0.0001) and ‘Picual’ (Pairwise Tests, p _‘Galega vulgar’ vs. ‘Picual’_ = 0.0001), and no significant differences between ‘Cobrançosa’ and ‘Picual’ (Pairwise Tests, p _‘Cobrançosa’ vs. ‘Picual’_ = 0.6195); in asymptomatic plants, individual pairwise comparisons between cultivars still showed significant higher fungal richness in ‘Galega vulgar’ compared to ‘Cobrançosa’ (Pairwise Tests, p _‘Galega vulgar’ vs. ‘Cobrançosa’_ = 0.0022) and ‘Picual’ (Pairwise Tests, p _‘Galega vulgar’ vs. ‘Picual’_ = 0.0001), and no significant differences between ‘Cobrançosa’ and ‘Picual’ (Pairwise Tests, p _‘Cobrançosa’ vs. ‘Picual’_ = 0.0559).

## 3. Discussion

In the present study, the fungal diversity associated with olive leaf spots in the presence of two major pathogens (*V. oleaginea* and *P. cladosporioides*), was investigated. Three different olive cultivars, with different susceptibilities to both pathogens, grown in the Alentejo region, were tested. Sampling was performed in spring, when lesions caused by *V. oleaginea* and *P. cladosporioides* are more evident.

Overall, 788 fungal isolates, from a total of 300 olive trees, were obtained and classified into 21 OTUs. Regardless of the health status or the cultivar, Ascomycota was clearly the most abundant Phylum, and Basidiomycota showed the lowest percentage of isolates. Interestingly, isolates from the Phylum Basidiomycota were not isolated in vitro and were only identified when direct total leaf DNA was used. This finding is possibly related to the media used and to the rapid colonization of fungi from Ascomycota that inhibit the growth of slow growing fungi, and demonstrate the importance of the use of these two methodologies to isolate a broader range of fungi. Despite that, proportions of these two Phyla obtained here are similar to other studies in olive as well as in other woody plants [28,31,32,33]. Within the Ascomycota, the class Dothideomycetes was the most representative followed by Leotiomycetes, Sordariomycetes, and Eurotiomycetes. The high incidence of the Dothideomycetes observed here is mainly due to the high number of isolates belonging to *V. oleaginea*, *Alternaria* sp., *Epicoccum* sp., and *Foliophoma* sp. as well as to *P. cladosporioides*. The Leotiomycetes identified were *Chalara* sp. and *Botrytis* sp. that, together with the above Dothideomycetes OTUs, represent nearly 80% of the fungal diversity found in this study. The Dothideomycetes is one of the largest classes and has shown to lead the diversity in the olive tree, as well as in other crops [28,31,32,33] while it comprises several plant pathogenic species, but also some antagonistic species. Within the Basidiomycota, the class Tremellomycetes was the most representative, mostly due to *Cryptococcus* sp. and *Bullera* sp.

The analysis of fungal richness in both health status, revealed significant higher values in the cultivar ‘Galega vulgar’ followed by ‘Cobrançosa’, which in turn did not show significant differences to ‘Picual’. In addition, ‘Galega vulgar’ also showed a higher fungal diversity when compared to the other cultivars, with the presence of five exclusive OTUs. The fact that over 80% of the Portuguese orchards are composed of trees from cv ‘Galega vulgar’, demonstrates the good establishment and adaptation of this cultivar in Portugal, and may partially explain the high values of fungal richness, and which are consistent with previous studies [28,29,30]. Other factors involving the chemistry of the plant, and certain types of phenolics in olive tissues have also been reported to determine the presence of some fungi and resistance to pathogens [34,35].

Concerning plant health status, the fungal communities revealed significant higher richness in symptomatic compared to asymptomatic plants in all cultivars, mostly due to the high number of isolates (230) from the pathogens *V. oleaginea* and *P. cladosporioides* that contribute to near 70% of the total isolates and that were not detected in any asymptomatic plants. However, when both pathogens were excluded from the analysis, only ‘Galega vulgar’ still maintained higher significant values of fungal richness in symptomatic plants when compared to asymptomatic plants, with ‘Cobrançosa’ and ‘Picual’ showing no significant differences between symptomatic and asymptomatic plants. Interestingly, despite the long latent period these two pathogens usually present, none of them was detected as latent, in asymptomatic leaves. This may be due to the fact that asymptomatic leaves were collected from trees with no apparent symptoms; meaning that pathogens may indeed not be present or they could be controlled by antibiosis or for space and nutrient competition by other endophytes. The incidence of *P. cladosporioides* and/or *V. oleaginea* in all symptomatic plants seems to confirm the role and shows the aggressiveness of these pathogens in the leaf spots. *P. cladosporioides* is the causal agent of olive cercosporiosis and *V. oleaginea* is the causal agent of peacock spot disease. This is not the case of other diseases, such as olive anthracnose, in which the causal agent, *Colletotrichum* sp., is frequently found as endophyte without causing any symptoms [30,31]. Interestingly, high levels of *V. oleaginea* were found in cv. ‘Galega vulgar’, known to be somewhat resistant to peacock disease, contrary to the other cultivars used, where it would be expected that *P. cladosporioides* dominates the pathogenic species.

Almost all of the remaining isolates found in symptomatic plants (27%) belonged to *Chalara* sp. and *Foliophoma* sp., being among the species most frequent in symptomatic trees. *Foliophoma* sp. has already been reported as the most abundant genera in trees infected with *V. oleaginea* [10]. Like *Chalara* sp., species within the *Foliophoma* genus have been described as saprophytes but also as pathogens causing dieback and shoot necrosis in olive [36,37,38]. No such symptoms were observed in the olive trees used in this study, however these two highly detected genera in symptomatic plants, can be a secondary effect of the disease, behaving as opportunistic pathogens and increasing the severity of the infection.

*V. oleaginea*, *P. cladosporioides*, *Chalara* sp., and *Foliophoma* sp. represent over 90% of the isolates detected in symptomatic plants. Differences on fungal composition between symptomatic and asymptomatic plants are clear, as seen in the PCO analysis (Figure 2). Although *Alternaria* sp. and *Epicoccum* sp. were present in both symptomatic and asymptomatic plants, in asymptomatic plants over 90% of the isolates are represented by these two OTUs, as opposed to 11% in symptomatic plants. Despite some *Alternaria* species having been reported to cause damages on olive [38,39,40,41], most species are saprophytic and/or endophytic. In this study no typical symptoms of pathogenicity caused by *Alternaria* sp. were seen in the olive trees used. The high incidence of these two genera in fungal communities inhabiting olive and other plants has been previously reported [30,42], however data comparing their presence with *V. oleaginea* and/or *P. cladosporioides* are inexistent. *Alternaria* and *Epicoccum* genera are both members of the Dothideomycetes, which are known to possess effective antagonistic mechanisms [42,43,44,45,46].

All remaining OTUs are represented by less than 3.5% each, and were either exclusive of symptomatic or asymptomatic plants, except for *Aureobasidium* sp. that appeared in both symptomatic and asymptomatic plants. This finding was expected since *Aureobasidium* sp. is one of the most abundant fungi present in the phyllosphere of several plants, including olive, where it has been isolated from the complex of saprophytic fungi that forms a sooty mold that may have an impact on olive fruit quality [30,47,48,49,50]. It is however often described as non-pathogenic and has also been reported as an effective bio-control agent due to its antagonistic activity against several pathogens [51,52,53,54]. *Botrytis* sp. isolates were only detected in symptomatic plants; it is a known pathogenic species, but no typical symptoms of this pathogen were seen in the sampled trees, suggesting that this pathogen is latent; its common occurrence as endophyte has been reported [42].

*Diaporthe* sp., *Arthrinium* sp., *Aspergillus* sp., and *Fusarium* sp. isolates were only detected in asymptomatic trees, and at low abundances (<2.5%). All these fungi have been reported as pathogens in a wide range of plant species, including olive [38,55,56]. The fact that they have been found in symptomless plants suggests that these fungi may colonize and establish latent infections. The analysis of the sequences of *Diaporthe* sp. and *Fusarium* sp. did not allow the identification to the species level, however, these genera comprise species that are pathogenic to olive, causing wilts, rots, cankers, and dieback symptoms [57,58]. *Fusarium* sp., *Aspergillus* sp. and *Diaporthe* sp. have been profiled for their volatile compounds and antifungal substances were detected [42,59,60]. They have already shown antagonistic activity against several pathogens namely *Colletotrichum* in olive and also in other plants.

In addition to the two pathogenic fungi focused in this study, another five OTUs were exclusive of symptomatic trees, although represented by less than 1% (*Bullera* sp., *Erythrobasidium* sp., *Neofusicoccum* sp., *Saccharata* sp., and *Sporobolomyces* sp.). From these five fungi, *Neofusicoccum* sp. have been described as endophytes or as plant pathogens that can cause infections on olive, causing shoot cankers, and dieback, and *Bullera* sp. comprise species with antagonistic functions. Nevertheless, currently available data on these species does not enable assumptions on the role of these fungi on olive to be supported.

In spite of the increasing number of studies concerning fungal diversity in olive, there is still more to learn about the associations they establish with known pathogens. To the best of our knowledge, this is the first study concerning fungal communities associated with these diseases. In conclusion, despite being performed in a localized area, this study showed a high fungal composition, similar to previous studies performed in different regions [28,31]. In this study, when olive plants from different cultivars, both asymptomatic and presenting leaf spots symptoms, were screened for fungal communities, two major groups of fungi were detected regardless of the cultivar: 90% of the isolates found in symptomatic plants belonged to V. oleaginea, *P. cladosporioides, Chalara* sp., and *Foliophoma* sp. and in asymptomatic plants, 90% of the isolates belonged to *Alternaria* sp. and *Epicoccum* sp. None of the dominant OTUs found in symptomatic trees were found in asymptomatic trees. This is very interesting, as *Alternaria* sp. and *Epicoccum* sp. seem to dominate the niches in asymptomatic plants.

Due to the known antagonistic role of these two genera, it would be interesting to study if there is any competitive action against these fungal plant pathogens, namely if their well establishment is preventing the development of putative later arriving species, such as *V. oleaginea* or *P. cladosporioides*.

It would be quite interesting to test if antagonistic activities exist and differ according to the order of infection of the several fungi, or if *Alternaria* sp. and *Epicoccum* sp. isolates, found in symptomless trees, present differences in genetic sequences or in gene expression from the *Alternaria* sp. and *Epicoccum* sp. isolates found in symptomatic trees. These data may be useful from a practical point of view with the aim of developing new more effective and less impacting means for disease management.

## 4. Materials and Methods

### 4.1. Sampling Collection

Leaf samples were collected in the spring of 2017 (from April to May) from olive trees belonging to three main cultivars ‘Galega vulgar’, ‘Cobrançosa’, and ‘Picual’, randomly selected within a field of 32 ha, near Monforte (Alentejo, south Portugal), where epidemic outbreaks of leaf spot disease occur on a yearly basis. The sampled orchard included programmed applications of fungicide and insecticide products such as copper hydroxide, trifloxystrobin, deltamethrin, and dimethoate.

Due to the difficulty in distinguishing peacock and cercospora leaf spots, both diseases were treated together; trees presenting leaf spots (either peacock or cercospora, or both) were considered ‘symptomatic’ and trees with apparently no symptoms were considered ‘asymptomatic’. All asymptomatic leaves were collected from trees with apparently no leaf spots in general. In addition, whenever possible, asymptomatic trees were chosen that had never presented these types of symptoms in the previous years. All olive trees sampled were 10 to 15 years old and of medium size. Sampling consisted in the collection of ca. 100 olive leaves around the whole tree at a height of ca. 2 m. A total of 300 samples (100 trees × 3 cultivars), half from symptomatic trees and half from trees with apparently no symptoms were taken. Each tree was treated as a sample.

To minimize the detection of epiphytic fungi as well as of microorganisms occasionally adherent to the leaf surface, leaf samples were rapidly disinfected with a sequence of 3 min immersions in 96% ethanol, 3% sodium hypochlorite solution, 70% ethanol, washed in ultra-pure water, and dried in sterile paper [30]. Leaves were stored at 4 °C until further processing. All samples were used for both fungal isolation (prior to DNA extraction) and for direct DNA extraction.

### 4.2. Fungal Isolation and DNA Extraction

Olive leaves were cut into small pieces of ca. 1 cm and placed on 9 cm diameter Petri dishes (five pieces per plate) containing Potato Dextrose Agar medium (PDA) (Merck, Darmstadt, Germany), at 25 °C ± 3 °C for 5 to 12 days. Colonies were then transferred to a new PDA plate for isolation. Mycelia from isolated fungi were ground in liquid nitrogen and stored at −80 °C until DNA extraction.

Both total DNA from samples and DNA from isolated fungi were extracted using the cetyltrimethyl ammonium bromide (CTAB) method [61], with some modifications [32]. For each sample, total DNA was extracted from ca. 500 mg of leaf material and ca. 250 mg of mycelium powder. DNA concentration was determined by using a Quawell Q9000 micro spectrophotometer (Quawell Technology, Beijing, China).

### 4.3. Fungal Molecular Identification

Fungal ITS regions were PCR amplified by using the primers ITS1 and ITS4 [62]. Reactions were performed in a total volume of 50 µL containing 30–80 ng of genomic DNA, 10 mM Tris-HCl (pH 8.6), 50 mM KCl, 1.5 mM MgCl2, 0.2 mM dNTPs (Fermentas, Waltham, MA, USA), 0.25 µM of each primer and 2.5 U DreamTaq DNA polymerase (Fermentas, Waltham, MA, USA). The amplification reaction was performed in a Thermal Cycler (Bio-Rad, Hercules, CA, USA) under the following temperature conditions: 95 °C for 2 min, 40 cycles of 95 °C for 30 s, 54 °C for 1 min, 72 °C for 1 min, and a final extension of 72 °C for 10 min. Amplified products were electrophoresed in 1% agarose gel and purified using DNA Clean & Concentrator (Zymo Research, Irvine, CA, USA). PCR products from DNA of isolated fungi were sequenced in both strands by Macrogen (Madrid, Spain). PCR products from direct DNA extraction were further cloned into pGEM-T easy vector (Promega, Madison, WI, USA) in accordance with the manufacturer’s instructions. Transformed E. coli JM109 cells were grown overnight at 37 °C at 175 rpm in LB medium (1% tryptone, 0.5% yeast extract and 0.5% NaCl, pH 7.5) with 100 mg/mL ampicillin. Plasmid DNA from 5 randomly selected colonies/samples was extracted using GenElute HP Plasmid Miniprep (Merck, Darmstadt, Germany) in accordance with the manufacturer’s instructions. Clones were sequenced in both strands by Macrogen (Madrid, Spain). Five ITS sequences/samples were determined. All sequences were analyzed using Bioedit Sequence Alignment Editor v.7.2.3 [63]. Taxonomic identification was done through the search for the closest sequence matches, using Basic Local Alignment Search Tools (BLAST) against the National Center for Biotechnology Information (NCBI) sequence database. Isolates were identified at species or genus level and classified as an Operational Taxonomic Unit (OTU).

### 4.4. Data Analysis

Univariate and multivariate analyses were performed to detect significant differences in total richness in the endophytic fungi in the two factors “Health status” and “Cultivar”. The statistical analyses of the data were performed using the PRIMER v6 software package [64] with the PERMANOVA add-on package [65]. The PERMANOVA analysis was carried out following the two factor design: Health status; “Symptomatic and Asymptomatic” (2 levels, fixed) and Cultivar: “Galega vulgar, Cobrançosa, and Picual” (3 levels, random). A two-way permutational analysis of variance (PERMANOVA) was applied to test the hypothesis that significant differences existed in the total richness in the fungi among the factors health status and cultivar. Total fungal data were square root transformed in order to scale down the importance of highly abundant endophytic fungi and therefore increase the importance of the less abundant ones in the analysis of similarity between communities. The PERMANOVA analysis was conducted on a Bray–Curtis similarity matrix [66]. The null hypothesis was rejected at a significance level <0.05 (if the number of permutations was lower than 150, the Monte Carlo permutation p was used). Whenever significant interactions in the effects of the factors were detected, these were examined using a posteriori pairwise comparisons, using 9999 permutations under a reduced model. All PERMANOVA procedures were also performed on the fungal richness data excluding both pathogenic fungi *V. oleaginea* and *P. cladosporioides*. The similarity in the endophytic fungi richness identified on each health status and cultivar was plotted by Principal coordinates analysis (PCO) using the Bray–Curtis similarity measure based on each of the two factors; health status and cultivar. The relative contribution of each fungus to the average of similarity between a priori defined groups; each health status and cultivar; was calculated using the one way-crossed similarity percentage analysis (SIMPER, cut-off percentage: 100%).

## Figures and Tables

**Table 1:** Fungal OTUs identified by SIMPER analysis that contribute most to the similarities at each; health status (Asymptomatic and symptomatic) and cultivar (‘Galega vulgar’, ‘Cobrançosa’ and ‘Picual’). Bold values highlight values higher than 0.05%.

Fungal OTUs	Asymptomatic	Symptomatic	Galega Vulgar		Cobrançosa		Picual	
	-	-	Asymptomatic	Symptomatic	Asymptomatic	Symptomatic	Asymptomatic	Symptomatic
	Similarity							
	37.40%	51.20%	48.09%	61.54%	38.18%	59.37%	30.78%	55.24%
*Alternaria* sp.	**62.81**	**0.75**	**56.18**	**0.92**	**77.48**	**0.74**	**49.28**	**0.22**
*Epicoccum* sp.	**29.11**	**3.37**	**37.42**	**4.2**	**10.1**	**3.22**	**43.82**	**1.41**
*Arthrinium* sp.	**2.06**	0.00	**1.81**	0.00	**2.35**	0.00	**1.33**	0.00
*Chaetomium* sp.	**1.83**	0.00	**0.97**	0.00	**3.35**	0.00	**0.93**	0.00
*Diaporthe* sp.	**1.68**	0.00	**0.86**	0.00	**1.28**	0.00	**2.71**	0.00
*Aspergillus* sp.	**1.10**	0.00	**0.19**	0.00	**5.28**	0.00	0.00	0.00
*Fusarium* sp.	**0.68**	0.00	**0.96**	0.00	**0.09**	0.00	**0.83**	0.00
*Nigrospora* sp.	**0.60**	0.00	**0.62**	0.00	**0.07**	0.00	**1.11**	0.00
*Aureobasidium* sp.	**0.14**	**0.21**	**0.99**	**0.02**	0.00	**0.12**	0.00	**0.42**
*Botrytis* sp.	0.00	**1.16**	0.00	**6.03**	0.00	**0.1**	0.00	0.00
*Bullera* sp.	0.00	<0.05	0.00	**0.23**	0.00	0.00	0.00	0.00
*Chalara* sp.	0.00	**16.99**	0.00	**22.73**	0.00	**34.46**	0.00	**0.73**
*Cryptococcus* sp.	0.00	**0.26**	0.00	**1.99**	0.00	0.00	0.00	0.00
*Erythrobasidium* sp.	0.00	<0.05	0.00	0.00	0.00	0.00	0.00	**0.21**
*Fusicladium* sp.	0.00	<0.05	0.00	**0.64**	0.00	0.00	0.00	0.00
*Neofusicoccum* sp.	0.00	<0.05	0.00	0.00	0.00	<0.05	0.00	0.00
*Foliophoma* sp.	0.00	**9.52**	0.00	**19.88**	0.00	0.00	0.00	**18.38**
*P. cladosporioides*	0.00	**27.14**	0.00	**16.49**	0.00	**23.97**	0.00	**33.36**
*Saccharata* sp.	0.00	<0.05	0.00	**0.14**	0.00	0.00	0.00	0.00
*V. oleaginea*	0.00	**40.42**	0.00	**26.52**	0.00	**37.35**	0.00	**45.27**
*Sporobolomyces* sp.	0.00	<0.05	0.00	**0.22**	0.00	0.00	0.00	0.00

**Table 2:** Details of the two-factor PERMANOVA test on the fungal dataset for the factors; “Health status” Asymptomatic and Symptomatic (2 levels, fixed) and “Cultivar” ‘Galega vulgar’, ‘Cobrançosa’ and ‘Picual’ (3 levels, random) for all variables analyzed. Bold values highlight significant effects and interactions (p < 0.05).

	Source of Variation	Degrees of Freedom	Sum of Squares	Mean Squares	Pseudo-F	Perms	P(perm)
Fungal	Health status	1	369990	369990	23.942	38	**0.0014**
richness	Cultivar	2	34596	17298	9.7174	9944	**0.0001**
-	Health status x Cultivar	2	30908	15454	8.6813	9948	**0.0001**
-	Residual	294	523360	1780	-	-	-
-	Total	299	958850	-	-		-
Fungal	Health status	1	914.28	914.28	0.71023	360	0.5397
richness*	Cultivar	2	14325	7162.5	53.698	9920	**0.0001**
-	Health status x Cultivar	2	2581	1290.5	9.6749	9941	**0.0001**
-	Residual	275	36681	133.39	-	-	-
-	Total	280	54692	-	-	-	-

* means PERMANOVA test on the fungal dataset excluding both pathogenic fungi *V. oleaginea* and *P. cladosporioides*.
